# Structural Racism, Place, and COVID-19: A Narrative Review Describing How We Prepare for an Endemic COVID-19 Future

**DOI:** 10.1089/heq.2021.0190

**Published:** 2022-05-12

**Authors:** Leah V. Estrada, Jessica L. Levasseur, Alexandra Maxim, Gabriel A. Benavidez, Keshia M. Pollack Porter

**Affiliations:** ^1^ Center for Health Policy, Columbia University School of Nursing, New York, New York, USA.; ^2^ Nicholas School of the Environment, Duke University, Durham, North Carolina, USA.; ^3^ School of Civil and Environmental Engineering, Georgia Institute of Technology, Atlanta, Georgia, USA.; ^4^ Department of Epidemiology and Biostatistics, University of South Carolina, Columbia, South Carolina, USA.; ^5^ Department of Health Policy and Management, Johns Hopkins Bloomberg School of Public Health, Baltimore, Maryland, USA.

**Keywords:** health equity, environmental justice, health systems and services, COVID-19, structural racism

## Abstract

**Background:**

Place is a social determinant of health, as recently evidenced by COVID-19. Previous literature surrounding health disparities in the United States often fails to acknowledge the role of structural racism on place-based health disparities for historically marginalized communities (i.e., Black and African American communities, Hispanic/Latinx communities, Indigenous communities [i.e., First Nations, Native American, Alaskan Native, and Native Hawaiian], and Pacific Islanders). This narrative review summarizes the intersection between structural racism and place as contributors to COVID-19 health disparities.

**Methods:**

This narrative review accounts for the unique place-based health care experiences influenced by structural racism, including health systems and services and physical environment. We searched online databases for peer-reviewed and governmental sources, published in English between 2000 and 2021, related to place-based U.S. health inequities in historically marginalized communities. We then narrate the link between the historical trajectory of structural racism and current COVID-19 health outcomes for historically marginalized communities.

**Results:**

Structural racism has infrequently been named as a contributor to place as a social determinant of health. This narrative review details how place is intricately intertwined with the results of structural racism, focusing on one's access to health systems and services and physical environment, including the outdoor air and drinking water. The role of place, health disparities, and structural racism has been starkly displayed during the COVID-19 pandemic, where historically marginalized communities have been subject to greater rates of COVID-19 incidence and mortality.

**Conclusion:**

As COVID-19 becomes endemic, it is crucial to understand how place-based inequities and structural racism contributed to the COVID-19 racial disparities in incidence and mortality. Addressing structurally racist place-based health inequities through anti-racist policy strategies is one way to move the United States toward achieving health equity.

## Introduction

Since the COVID-19 pandemic was first reported in the United States in January 2020, as of March 1, 2022, more than 79 million infections and more than 951,081 COVID-19-related deaths have been reported.^1^ The impact of COVID-19 has varied geographically across the United States and has disproportionately affected historically marginalized communities (i.e., Black and African American communities, Hispanic/Latinx communities, Indigenous communities [i.e., First Nations, Native American, Alaskan Native, and Native Hawaiian],^2^ and Pacific Islanders).^3,4^

Black and African Americans account for 35% of the U.S. population, and while only 22% of U.S. counties have more Black and African American populations than the national average (defined as greater than 13%), these same counties accounted for 47% of cases and 54% of COVID-19 deaths nationwide.^5^ Contrastingly, counties with the highest proportion of White residents accounted for the lowest incidence of COVID-19.^6^ The COVID-19 pandemic has highlighted how race and place intersect to affect health outcomes, with historically marginalized communities vulnerable to increased COVID-19 incidence and death.

Place is critical to health: where one lives, what one has access to, and what one is exposed to influences health outcomes. Although previous literature has acknowledged the role of place in health disparities,^7–10^ only recently has structural racism been identified as a major contributor to health disparities, including those observed from COVID-19.^6,11–13^ Structural racism is defined as the ways in which “societies foster mutually reinforcing inequitable systems that reinforce discriminatory beliefs, values, and distribution of resources.”^14^ Discriminatory practices and policies have been created, by design, to embed inequities for historically marginalized communities.^15^

However, when structural racism has been acknowledged as a significant contributor to COVID-19 disparities,^16^ the connection to place is missing. Addressing this gap in the literature is necessary to fully address place-based health disparities,^8^ which result from structural racism. This narrative review names and examines the intersection between structural racism and place as contributors to COVID-19 health disparities. To address disparities in COVID-19 incidence and death, it is crucial to understand why historically marginalized communities have been and will continue to be at risk unless anti-racist policies are created.

## Methods

### Conceptual framework

**FIG. 1.**
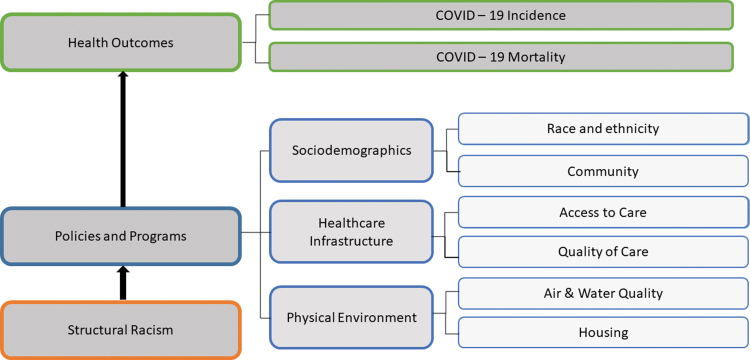
Modified conceptual model.

A modified County Health Rankings Model, created by the County Health Rankings and Roadmaps program, a collaboration between the Robert Wood Johnson Foundation and the University of Wisconsin Population Health Institute,^17^ guided this narrative review (Fig. 1). The County Health Rankings Model illustrates how sociodemographic factors, health care infrastructure, and environmental factors are associated with population health.

Our modified model illustrates how structural racism influences place (i.e., defined as a confluence of sociodemographic factors, health care infrastructure, and the physical environment), which contributes to adverse COVID-19 outcomes (i.e., increased incidence and death). Given our focus on structural racism and place, we did not include individual factors (e.g., diet and exercise, tobacco use), which were in the original model. Guided by this model, we aim to narrate how multiple factors related to place are influenced by structural racism, and how this has led to increased COVID-19 incidence and death among historically marginalized communities.

In this narrative review, we refer to place as the physical location, community, and/or neighborhood where people reside and the structural resources available to them, such as health systems and services and physical environment factors. We argue that structural racism causes place-based health inequities that are demonstrable in historically marginalized communities throughout the United States. Our review focuses on two key aspects of place—*health services and systems*, which includes access to and quality of care, and *physical environment*. For this review, we limit the physical environment to the quality of outdoor air, drinking water, and housing (e.g., neighborhood and physical building where people reside).

### Synthesis of findings

Our narrative review is structured in four parts. First, we present the role that structural racism has played for centuries in the United States to provide context for how it has manifested during the COVID-19 pandemic. Herein review, we use “historically marginalized communities” as a collective term, as defined above, and name specific racial and ethnic groups when the studies cited refer to a specific population. We also refrain from using race interchangeably with socioeconomic status^18^ since these are variables confounded by each other.^19,20^ The likelihood that historically marginalized communities reside in areas of concentrated poverty is due to structural racism stemming from not only individual bias but also policies deeply embedded in society that have created separate and unequal living conditions.^14,21^

Second, we briefly summarize the literature searched from online databases (e.g., PubMed) for peer-reviewed literature in English from the United States as well as governmental and organizational reports. We included the literature that explored historically marginalized communities and place in the context of health systems and services (e.g., hospitals, primary care, nursing homes, pharmacies) and the physical environment (e.g., outdoor air quality, drinking water quality, and housing). Included literature went through ascendency and descendancy reviews for articles of interest. Our search was limited to publications between 2000 and 2021.

Third, we describe factors from health services and systems and the physical environment through the lens of COVID-19 and structural racism. We explore the link between the historical trajectory of structural racism and current COVID-19 health outcomes for historically marginalized communities.

Finally, drawing on the literature we reviewed, we discuss the need for anti-racist policies to prevent further COVID-19 risk/vulnerability and advance health equity for historically marginalized communities. These policies are essential to eliminate currently the structurally racist place-based health disparities that have negatively impacted historically marginalized communities.

## Results

### Historical context of structural racism and place in the United States

More than 99% of neighborhoods in the United States are racially segregated,^22^ which exposes constituents to certain iniquities that are grounded in place.^23^ Racially segregated neighborhoods are the result of structural racism.^24^

Since the 17th century, Black and African American communities have been economically deprived and impoverished with poorer health outcomes.^14^ Structural racism and social control were exercised over formerly enslaved populations and have persisted through continued segregation.^14^ Historically marginalized communities have experienced housing segregation due to policies instituted at every level of the U.S. government. For example, practices such as redlining created segregated communities from the 1930s,^25^ which prohibited sale of land and homes to Black and African Americans, preventing them from building generational wealth and continuing the cycle of generational poverty.^18^

### Health systems and services as a place-based issue

Access to quality health care systems and services is a critical contributor to health inequities in the United States. The Civil Rights Act of 1964 removed legal segregation in health care settings, but health care segregation continues.^26,27^ Unsurprisingly, this health care segregation often mirrors the racial residential segregation of historically marginalized communities.^28^

Historically marginalized communities are often left with fewer options to receive preventative health care due to a lack of access to community health centers. Additionally, place-based barriers to timely health care have been associated with a community's distance to a health care center and their reliance on public transit.^29^ Lack of or inaccessibility of transportation has been associated with less health care utilization, lack of regular medical care, and missed medical appointments, and these barriers to medical care are greater among historically marginalized groups compared with Whites.^29^

Individuals from historically marginalized communities have a decreased likelihood of having a primary care provider, an increased likelihood of having unmet health needs, and a decreased likelihood of receiving preventative care.^30^ This lack of health care access has contributed to disproportionate vulnerability and high rates of cancer and obesity-related chronic diseases for Black and African Americans,^31^ chronic conditions among Indigenous,^32^ and cardiovascular disease among Hispanics^33,34^ as examples. This disproportionate chronic condition vulnerability contributed to subsequent COVID-19 vulnerability.^35^

#### Hospital access

Historically marginalized communities often have high levels of concentrated poverty and high uninsured rates. This has resulted in local hospital closures, especially of safety-net hospitals, which often serve as the primary source of health care for these individual communities.^36^

Safety-net hospitals serve a high percentage of Medicaid (reimbursing at a far lower rate than private health insurance) and uninsured patients. Both income and racial residential segregation are negatively associated with physician participation in Medicaid.^37^ While safety-net hospitals have improved access to care for historically marginalized communities,^38^ these hospitals have suffered financially due to low insurance reimbursement.^39^ Consequently, safety-net hospitals have decreased costs by furloughing or laying off employees and ultimately closing.^36,39^ Closures disrupted health care, particularly for those seeking preventative care, mental health services, and hospitalizations for births.^40^ Furthermore, neighboring emergency departments were understaffed to meet the increased need and influx of new patients.^39^

In a study that examined the role of racial residential segregation and community-level poverty on trauma deserts (i.e., areas with a travel distance greater than 8.0 km to the nearest adult level I or level II trauma center) in Chicago, Los Angeles, and New York City found that the majority of Black and African American neighborhoods were more likely to be in a trauma desert in all three cities, whereas the majority of Hispanic communities more likely to be in a trauma desert in Chicago but not in Los Angeles or New York City.^41^ The researchers attributed this difference to the history of structural racism, which has manifested in place-based issues that have created higher inequality over a longer period for Black and African Americans.^41^ Similarly, a 2021 report by the Office of Inspector General reported that COVID-19 exacerbated long-standing challenges in health care delivery particularly for historically marginalized communities.^42^

#### Community health centers access

Before the COVID-19 pandemic, the number of community health centers and federally qualified health centers had expanded and were often located in historically marginalized communities^43^; yet there remained more than 7,000 Primary Care Health Professional Shortage Areas, with a deficit of more than 15,000 health care providers, affecting 83 million patients.^44^ Additionally, the quality of health services at federally qualified health centers has sometimes been unfavorable. Historically marginalized communities have reported perceived racial discrimination, undertreated pain,^45^ and overall patient dissatisfaction, often attributed to poor provider communication, at federally qualified health centers.^46^

There are an estimated 3,439 Medically Underserved Areas nationwide, affecting 14 million individuals.^47^ These designations indicate too few primary care providers, high poverty, or a high population of older adults.^47^ Notably, there is a lack of information on the racial and ethnic demographics of these areas. Over time, community health centers and federally qualified health centers have limited the uninsured care provided,^27^ further limiting access. At the height of the COVID-19 pandemic during lockdowns, these community health centers experienced sharp decreases in clinic visits, which led to 15% of sites closing in May 2020 and others at risk for closing due to increased costs with few visits.^48^

#### Nursing homes

Inequities in access to quality health care persist across the life span. There has been an increase of Black and African American and Hispanic older adults in nursing homes and a decrease in White older adults who often have access to more long-term care options.^49,50^ Nursing homes provide long-term medical care services, including nursing care and continued supervision.^51^ Black and African American residents often have multiple, complex, chronic conditions while approaching the end of life but receive worse end-of-life care than White older adults.^52^ Despite the increased need for nursing home care among older adults, nursing homes are costly, and to qualify for Medicaid, individuals often need to “spend down” assets to receive coverage.^53^

Nursing homes serving higher concentrations of Black and African American residents have reported lower staffing levels, higher rates of health-related deficiencies, lower Centers for Medicaid and Medicare quality star ratings, lower influenza vaccination rates, and serve a higher percentage of Medicaid residents.^54–56^ These nursing homes are typically located in racially segregated communities, highlighting the impact of place in historically marginalized communities.^55,56^ Unsurprisingly, at the height of the COVID-19 pandemic, ∼40% of all COVID-19 related deaths^57^ (actual percentage expected to be higher^58^) were in nursing homes with higher proportions of Black and African American and Hispanic residents.^59^

#### Pharmacy access

Individuals from historically marginalized communities share a disproportionate burden of chronic conditions across the United States that often require pharmacological treatment. Yet pharmacy deserts (i.e., lack of geographic accessibility to pharmacies) are more prevalent in historically marginalized communities. Previous work found that racial residential segregation was associated with pharmacy deserts.^60^ Pharmacy deserts are often in communities with higher concentrations of Black and African American and Hispanic residents, less vehicle ownership, higher concentration of individuals living below the federal poverty level, and fewer health care providers serving the area.^61^

In a study examining the 30 most populated U.S. cities, pharmacy deserts were disproportionately identified in communities with higher concentrations of Black and African American or Hispanic individuals compared with White or more diverse communities. These communities had more pharmacy closures, further limiting access to health care needs.^62^ Mail order pharmacy has been suggested as a means to counter pharmacy deserts, but most users tend to be White older adults with higher income and greater education.^63–65^ Access to community pharmacies was critical to receive routine care through medication dispensation and provide accessible vaccine disbursement in familiar and convenient locations,^66^ particularly important during the COVID-19 pandemic.

#### Access for rural communities

Health system and service inequities are equally present in rural historically marginalized communities. These communities in rural settings are often poorer, with worse health status compared with their urban counterparts.^67^ For U.S. Indigenous people who live in rural areas and have little access to specialty care due to being underinsured, both a lack of local specialty providers and transportation barriers influence their health outcomes.^68^ Rates of rural hospital closures are significant, with more than 100 in the past decade.^69^ Rural nursing homes have lower Centers for Medicaid and Medicare quality star ratings and lower staffing, and increased rurality is associated with lower quality of care.^70^

Rural pharmacy deserts have also been concentrated in historically marginalized communities.^71^ Furthermore, rural historically marginalized communities have been negatively associated with access to a usual source of care.^72^ Lack of or inaccessibility of transportation has been associated with less health care utilization, lack of regular medical care, and missed medical appointments, and these barriers to medical care are greater among historically marginalized groups compared with Whites.^29^ Unsurprisingly then, COVID-19-related racial disparities still persisted in rural settings^73^ for all the same aforementioned reasons.

### Physical environment as place-based issue

Historically marginalized communities are most affected by environmental pollution in the United States^74–76^ and more likely to live in more polluted areas.^77^ In 2007, the United Church of Christ reported that historically marginalized communities are more likely to be surrounded by toxic exposures from facilities compared with White communities.^74^

Black and African American, middle-class Americans are more likely to experience greater exposure to environmental pollutants, such as air pollution, water pathogens, industrial chemical pollution, and potentially toxic elements such as heavy metals than lower-class Whites,^78^ noting that these injustices are not tied to solely poverty.^78,79^ Superfund sites and “hyper-polluters” (i.e., U.S. industrial facilities)^80^ have been identified as disproportionately exposing historically marginalized communities to chemical releases and pollutants,^81^ and historically marginalized communities are more impacted than White communities by producers who disproportionately create pollution.^80^

#### Air pollution

The association between COVID-19 incidence and air quality has been noted extensively.^82,83^ For example, air pollution increases the risk of proposed COVID-19 risk factors, such as hypertension and diabetes.^16,84^ Historically marginalized communities are more likely to live in neighborhoods or areas with higher levels of air pollution.^85–87^ The American Lung Association found that compared with White communities, historically marginalized communities were 61% more likely to live in a county with a failing grade for at least one pollutant measured (short-term PM_2.5_, long-term PM_2.5_, and O_3_), and over three times more likely to live in a county that had a failing grade for all three pollutants measured.^88^

In a 2019 study on inequities in pollution levels, Black and Hispanic individuals were estimated to bear more than 50% excess air pollution in their home communities relative to how much pollution they produce, whereas Whites were estimated to experience 17% less air pollution compared with what they produce.^89^ In Detroit, Hispanic populations experience an increased burden of industrial pollution from living nearby industrial facilities such as from coal-fired power plants, cement facilities, or incinerators.^90^ Industry's tendency to cluster together geographically (e.g., oil and gas production, cement and chemical manufacturing, metal processing, mining, petroleum refineries, pulp and paper) means that historically marginalized communities generally live in those areas.^90^

Transportation creates a significant source of air pollution, particularly in cities. On roads with higher density of traffic, greater levels of PM_2.5_ have been noted in cities such as Detroit.^91^ The highest concentration of these pollutants is usually found nearer to the major roadways, and residence in these locations is exposed to elevated levels of these pollutants. Carbon dioxide (CO_2_) emissions from road travel were found to decrease as the concentration of Whites increases in a county, whereas counties with higher Black and African American populations were found to emit more CO_2_.^92^

As infrastructure improved in Boston neighborhoods with higher concentrations of Black and African American and Hispanic populations, source emissions (such as from more transportation infrastructure or higher traffic counts) increased, causing higher PM_2.5_ and NO_2_ exposures. Across Massachusetts, air pollution inequalities have increased in historically marginalized communities even as pollution levels have decreased across the state overall.^93^ Air pollution has been associated with increased risk of proposed COVID-19 risk factors (i.e., hypertension and diabetes) and has also been associated with increased risk of cancer among Hispanic populations compared with Whites.^94^

With cancer listed by the U.S. Centers for Disease Control as a risk factor for COVID-19,^95^ these impacts are doubly relevant in their disparities. Higher PM_2.5_ and toxic air pollution risk levels were associated with race and COVID-19 mortality rates in Louisiana. Notably, parishes in Louisiana with higher concentrations of Black and African American residents bare a larger burden of air pollution than parishes with lower concentrations of Black and African American residents.^96^

#### Water quality

COVID-19 vulnerability may be impacted by the presence of drinking water contaminants, such as metals like lead, due to their ability to alter immune system responses.^97^ Historically marginalized communities may have less access to clean drinking water and are more likely to have access to polluted drinking water than White communities.^98^ For example, these communities are more likely to live in neighborhoods with higher levels of lead pollution in their water.^78^ A Michigan state government-appointed civil rights commission reported that the slow response to the Flint water crisis was a direct result of the structural racism woven into the infrastructure, housing, employment, regionalization, and industry of Flint and the surrounding area.^99^

Historically marginalized communities, including some Indigenous populations, lack piped water or only have access to water supplies with pathogen contaminations.^100–103^ Without access to clean water for sanitation and hygiene, basic COVID-19 mitigation strategies are compromised in communities without access to quality water supplies. Nearly half of all Native American households on Indian Reservations in the United States are believed to not have access to clean water or adequate sanitation.^104^ Without access to water that is safe, Native Americans are unable to adequately or safely hand wash, prepare food, or drink water, all activities necessary for both survival and COVID-19 protection.^104^

In Alabama, safe water access is limited in historically marginalized communities, such as in Lowndes County, where the soil has low permeability.^105^ These lowlands in Lowndes County were prone to flooding but were the only area where Black and African American residents could acquire or afford land in the early 1900s, whereas White residents were able to afford higher elevation land with better drainage.^106^ Residents of Lowndes County reported raw sewage pooling near where people live due to problems with sewage disposal and septic systems.^107–109^

Although more than 99% of the U.S. population receive piped drinking water, historically marginalized communities experience inequities in access or potability of water received where they live.^110–115^ An investigation of well water in Black and African American communities in North Carolina demonstrated poorer quality drinking water than those on municipal water systems.^116^

Generally, the COVID-19 standardized mortality ratio for Native Americans has been higher than any other historically marginalized community, with increased risk associated with one's reservation status.^117^ COVID-19 cases reported on reservations were more likely to be associated to homes without running water more than any other household variables investigated.^118^ Insecure and compromised domestic water access in the United States is not only an issue with the physical environment but also a housing issue that is directly related to structural racism throughout the United States,^118^ particularly in cities.^98^

#### Housing

Racially segregated neighborhoods are the result of structural racism,^24^ and residential segregation may also increase exposure to COVID-19 due to factors such as housing density.^16^ Counties in the United States with higher percentages of multi-unit households had higher COVID-19 incidence.^119^ Household crowding was associated with COVID-19 incidence, suggesting that multi-unit housing, building shared spaces, or close proximity to neighbors may be risk factors for COVID-19.^13,119^ Decades of race-based segregation has created dense neighborhoods and overcrowded buildings in many historically marginalized communities, possibly contributing to higher rates of communicable diseases, including COVID-19, compared with less dense neighborhoods.^120,121^

Additionally, asthma diagnoses have been independently associated with poor housing quality.^122^ This poor housing quality, defined as “cracks in walls/floors/windows, broken plumbing, or exposed wires” may increase the exposure to allergens that exacerbate asthma as well.^122^ Since asthma is associated with increased risk of COVID-19 incidence and mortality,^95^ poor housing quality may contribute to COVID-19 incidence and mortality disparities in historically marginalized communities.

## Discussion

### Implications for public health and policy

To achieve health equity for historically marginalized communities, policy and practice must dismantle structural racism. Disparities in COVID-19 incidence and mortality^119,120^ are associated with place-based racist policies (including laws, rules, regulations, and guidelines that govern people) that have perpetuated health disparities.^119,120,123^ To note, historically marginalized communities should not have to leave their community for equitable health systems and services, and a safe physical environment.

Achieving health equity should not be dependent on one's place. Anti-racist policies^124,125^ are necessary to not only acknowledge how racism has shaped policies and practices historically but also continue to shape our surroundings, including where we live. Historically marginalized communities have experienced increased incidence and death from COVID-19, and this narrative review has detailed how place-based structural racism allowed these health inequities to occur.

We recommended the development and implementation of anti-racist policies to prevent these health inequities from occurring again. Examples of anti-racist policies that address place-based inequities are dedicated funding to increase primary care, hospital, and pharmacy access in historically marginalized communities; or policies to protect against place-based environmental injustices such as requiring ongoing air quality and water quality testing and monitoring in areas where historically marginalized communities reside. As these anti-racist place-based policies are implemented, it will be essential to build effective partnerships between institutions and communities. Furthermore, embedding accountability measures to ensure health equity is being advanced and not exacerbated by these anti-racist policies will also be key.

## Conclusion

This narrative review broadly summarized how place highlights barriers due to structural racism, with a focus on the COVID-19 pandemic. As COVID-19 becomes endemic, it is crucial to understand how place-based inequities and structural racism contributed to the COVID-19 racial disparities in incidence and mortality. While a significant amount of research exists on place-based inequities, they often fail to mention structural racism's contribution. Implementing policies that address place-based health inequities are critical as COVID-19 becomes endemic. Now is the time to reconstruct our country's legacy on health equity as structural racism has caused severe and disproportionate health effects for historically marginalized communities.

## Abbreviation Used

CO_2_: carbon dioxide
